# Nosocomial infections in the surgical intensive care unit: an observational retrospective study from a large tertiary hospital in Palestine

**DOI:** 10.1186/s12879-023-08677-z

**Published:** 2023-10-13

**Authors:** Banan M. Aiesh, Raghad Qashou, Genevieve Shemmessian, Mamoun W. Swaileh, Shatha A. Abutaha, Ali Sabateen, Abdel-Karim Barqawi, Adham AbuTaha, Sa’ed H. Zyoud

**Affiliations:** 1 Infection Control Department, An-Najah National University Hospital, Nablus, 44839 Palestine; 2 Department of Medicine, College of Medicine and Health Sciences, An-Najah National University, Nablus, 44839 Palestine; 3 Department of Internal Medicine, An-Najah National University Hospital, Nablus, 44839 Palestine; 4 Department of General Surgery, An-Najah National University Hospital, Nablus, 44839 Palestine; 5 Department of Biomedical Sciences, Faculty of Medicine and Health Sciences, An-Najah National University, Nablus, 44839 Palestine; 6 Department of Pathology, An-Najah National University Hospital, Nablus, 44839 Palestine; 7 Department of Clinical and Community Pharmacy, College of Medicine and Health Sciences, An-Najah National University, Nablus, 44839 Palestine; 8 Poison Control and Drug Information Center (PCDIC), College of Medicine and Health Sciences, An-Najah National University, Nablus, 44839 Palestine; 9 Clinical Research Center, An-Najah National University Hospital, Nablus, 44839 Palestine

**Keywords:** Nosocomial infections, Surgical intensive care unit, SICU, Healthcare-associated infection, HAI

## Abstract

**Background:**

Nosocomial infections or hospital-acquired infections are a growing public health threat that increases patient morbidity and mortality. Patients at the highest risk are those in intensive care units. Therefore, our objective was to provide a pattern analysis of nosocomial infections that occurred in an adult surgical intensive care unit (ICU).

**Methods:**

This study was a retrospective observational study conducted in a 6-bed surgical intensive care unit (SICU) at An-Najah National University Hospital (NNUH) to detect the incidence of nosocomial infections from January 2020 until December 2021. The study group included 157 patients who received antibiotics during their stay in the SICU.

**Results:**

The incidence of nosocomial infections, either suspected or confirmed, in the SICU was 26.9% (95 out of 352 admitted patients). Pneumonia (36.8%) followed by skin and soft tissue infections (35.8%) were the most common causes. The most common causative microorganisms were in the following order: *Pseudomonas aeruginosa* (26.3%), *Acinetobacter baumannii* (25.3%), *extended-spectrum beta lactamase (ESBL)-Escherichia coli* (23.2%) and *Klebsiella pneumonia* (15.8%). The average hospital stay of patients with nosocomial infections in the SICU was 18.5 days.

**Conclusions:**

The incidence of nosocomial infections is progressively increasing despite the current infection control measures, which accounts for an increased mortality rate among critically ill patients. The findings of this study may be beneficial in raising awareness to implement new strategies for the surveillance and prevention of hospital-acquired infections in Palestinian hospitals and health care centers.

## Background

Nosocomial infections (NIs) are infections acquired after 48 h of hospital admission [1], and they continue to be a significant problem in hospitalized patients across the globe [2, 3]. Patients are prone to develop various infections while receiving healthcare services for another condition in any healthcare department [4]. Despite the ongoing progression and development in hospital care, the prevalence of infections continues to increase [5].

Every day, one out of every 31 hospitalized patients is afflicted with a healthcare-associated infection (HAI) [6], which can be caused by various microorganisms that lead to different types of nosocomial infections, such as respiratory tract infections (RTIs), urinary tract infections (UTIs), skin and soft tissue infections (SSTIs), bloodstream infections (BSIs), and surgical site infections (SSIs) [7, 8].

Individuals who are hospitalized in the surgical intensive care unit (SICU) have a higher likelihood of developing nosocomial infections than those who are admitted to other wards within the hospital. While only 6% of patients develop infections in hospital wards, the overall risk of nosocomial infections is 18% in the SICU [9]. The rate of NIs is currently estimated to be 5–15% in developed countries compared to 25% in less developed countries [10]. Many predisposing factors increase the risk in these patients: patient health status (advanced age, immunosuppression, or chronic diseases), the indication of admission to the SICU (surgery, trauma, burns), invasive procedure (mechanical ventilation, central venous catheter, urinary catheter), and treatment-related factors (duration of preoperative hospitalization, type of surgery, need for blood transfusion, immunosuppressive therapy, recumbent position, and length of hospital stay) [1, 11].

Concerning surgical ICU infections, NIs are a major concern because they contribute to increased morbidity and mortality rates [7, 8, 12–14]. In addition, NIs can cause postoperative complications, extend hospital stays by up to 13 days, and increase healthcare costs [15–17]. The presence of NIs has a detrimental effect on both patient and healthcare worker safety [4]. To address the issue of nosocomial infections in the surgical ICU, it is essential to understand the microbiological profile of the microorganisms responsible for these infections, which can aid in the development of effective strategies to reduce the prevalence of nosocomial infections and minimize their impact on patient outcomes [18, 19].

Analyzing NI profiles in a specific SICU helps healthcare providers identify key bacteria causing these diseases. This aids in assessing bacterial susceptibility to antibiotics and understanding spread factors. This information is crucial for effective infection control strategies, encompassing hand hygiene, protective gear use, and thorough cleaning. Moreover, comprehending infection composition assists in choosing suitable antibiotic treatments, considering pathogen sensitivity to medications. This can help limit the emergence of antibiotic resistance and ensure that patients receive the most effective treatment. In Palestine, limited data have been reported regarding the incidence or prevalence of NIs and their risk factors among patients admitted to surgical ICUs. In addition, no previous research on NIs was performed in Nablus. This study aims to determine the incidence of NIs in the surgical ICU at An-Najah National University Hospital throughout 2020–2021.

## Methods

### Study design

A retrospective study was conducted in a tertiary care hospital in Palestine, An-Najah National University Hospital (NNUH). We reviewed the medical files and records of all patients admitted to the SICU who received antibiotics throughout their stay in the SICU between the start of 2020 and the end of 2021. There was no follow-up for any exposure, so no cohort or case‒control study was needed. In this study, we aimed to study the types of hospital-acquired infections, antibiotics used, patient characteristics and outcomes.

### Ethical considerations

Approval for all aspects of the study protocol, which included accessing and utilizing patient clinical information, was granted by the *Institutional Review Boards (IRBs) of An-Najah National University*. The confidentiality of the data and information was maintained and restricted to clinical research purposes. Patient identifiable information was not disclosed, and numerical codes were used in place of patient names.

### Study population

Patients who had nosocomial infections during their stay in the SICU of this tertiary care center were our targeted population in this study. Inclusion criteria: (1) adult patients of 18 years or older and (2) infections that occurred at least 48 h after admission according to the Centers for Disease Control and Prevention (CDC) criteria. The exclusion criteria were as follows: (1) pediatric patients and (2) patients who presented to the SICU with proven infections.

### Setting

The study was carried out at An-Najah National University Hospital, which is a tertiary academic hospital with a capacity of 135 beds. The SICU is a closed unit that is divided into two sections. The first is four beds, and the other is 2 beds for patients who need transmission-based precautions.

### Sample size

This study collected data from SICU patients admitted who received antibiotics during their stay in the SICU for either confirmed or suspected nosocomial infections between January 2020 and December 2021. Fifteen patients were excluded due to incomplete medical records. Therefore, data were collected, studied, and analyzed for 157 patients who were given antibiotics in the SICU during this period. Of these, 95 patients were given antibiotics to treat suspected or confirmed NIs. It is worth noting that a total of 352 patients were admitted to our SICU during the study period.

### Data collection

The records of patients who were admitted to the SICU and received antibiotics during their surgical ICU stay were reviewed. We separated those who had received these antibiotics from those who had infections upon presentation. The data were collected and entered into a data collection form that included the following sections:

Section 1: Demographic and clinical characteristics data, including age, sex, admission diagnosis, comorbidities, complications such as septic shock, length of stay, and patient outcome.

Section 2: Data regarding the source of nosocomial infection and devices introduced to patients.

Section 3: Isolated pathogen types (gram-negative, gram-positive, or Candida).

### Statistical analysis

The data underwent coding and categorization before being input into version 21.0 of the IBM-SPSS software. Sociodemographic and clinical data were analyzed using descriptive statistics such as frequency, percentage, mean, and standard deviation.

## Results

### Demographic and clinical characteristics

The demographic factors of SICU patients who were classified as having HAI (whether suspected or confirmed) were studied, including sex and age. Of these patients, 68 (71.6%) were males, and 27 (28.4%) were females, with a mean age of 57.69 ± 17.82.

The most prevalent comorbidities among these patients were hypertension and diabetes mellitus, with frequencies of 50.5% and 46.3%, respectively. Other comorbid illnesses are illustrated in Table 1. The main cause of admission to the SICU varied among patients. The most common causes of admission to the SICU were neurosurgery in 50 cases (31.85%), general surgery in 49 cases (31.15%), and trauma in 12.7% of patients.

**Table 1:** Demographic and clinical characteristics of patients with nosocomial infections

Variable		Total n (%) n = 157 (100%)	With nosocomial Infection N = 95 (61%)		Without nosocomial infection N = 62(39%)
**Age (years), mean ± SD**		55.52 ± 17.88	57.69 ± 17.820		52.18 ± 17.602
**Sex**					
Male		110 (70.1)	68 (71.6)		42 (67.7)
Female		47 (49.9)	27 (28.4)		20 (32.3)
**Comorbidities**					
Hypertension Diabetes mellitus Malignancy		78 (49.7) 69 (43.9) 21 (13.4)	48 (50.5) 44 (46.3) 15 (15.8)		30 (48.4) 25 (40.3) 6 (9.7)
Chronic kidney disease Others		19 (12.1) 38 (24.2)	14 (14.7) 20 (21.1)		5 (8.1) 18 (29)
Source of infection*					
Undetermined/suspected		50 (31.8)	7 (7.4)		43 (69.4)
Skin and soft tissue infection		39 (24.8)	34 (35.8)		5 (8.1)
Urinary tract infection Pneumonia		37 (23.6) 39 (24.8)	32(33.7) 35 (36.8)		5 (8.1) 4 (6.8)
Intraabdominal infection Bloodstream infection		23 (14.6) 26 (16.6)	19(20) 26 (27.4)		4 (6.5) 0 (0)
Meningitis		11 (7)	9 (9.5)		2 (3.2)
**Septic shock**		85 (54.1)	67 (70.5)		18(29)
**Length of stay (days), mean ± SD**		13.46 **±** 14.88	18.53 **±** 16.339		5.69 **±** 7.339
**Outcome**					
Discharged		103 (65.7)	52(54.7)		51(82.2)
Died		54 (34.4)	34 (45.3)		11 (17.7)

Approximately 67 (70.5%) of the patients with NIs developed septic shock. The average hospital stay for patients diagnosed with these infections in the SICU was 18.53 ± 16.33. Regarding the outcome of hospital care in the SICU with nosocomial infections, 52 (54.8%) patients were discharged. The overall mortality rate of patients diagnosed with HAI in the SICU was 34.4%.

Of the proven nosocomial infections, the most frequently reported were pneumonia (36.8%), skin and soft tissue infections (35.8%) and urinary tract infections (33.7%). Bloodstream infections were attributed to 27.4% of all NIs in the studied patients. It should be noted that more than one source of infection was often discovered in these patients. All these details are shown in Table 1.

Our studied patients had different invasive device placements. Most of the patients (83.2%) had an endotracheal tube, 61 patients (64.2%) had a urinary catheter, 41 patients (43.2%) had a central line, and many patients required other devices to a lesser extent, as shown in Table 2.

**Table 2:** Devices inserted into patients diagnosed with nosocomial infections

Variable	Total n = 157 (100%)*	With nosocomial Infection N = 95 (61%)*	Without nosocomial infection N = 62 (39%)*
Endotracheal tube	123 (78.3)	79 (83.2)	44 (71%)
Urinary catheter	98 (62.4)	61 (64.2)	37(59.7)
Central line	56 (35.7)	41(43.2)	15 (24.2)
Nasogastric tube	20 (12.7)	17(17.9)	3(1.8)
Percutaneous endoscopic gastrostomy tube	10 (6.4)	8(8.4)	2 (3.2)
Drains Chest tube	10 (6.4) 9(5.7)	7 (7.4) 7 (7.4)	3 (4.8) 2(3.2)
Others	5(3.2)	5(5.3)	0 (0)

*total exceeds 100% as data are overlapping due to multiple devices inserted into patients

### Microbial profiles of patients with nosocomial infections in the SICU

Gram-negative organisms were more prevalent than gram-positive organisms in the tested clinical samples. Of the culture-confirmed nosocomial infections, gram-negative organisms were reported in 115 samples representing 15 different pathogens, with *P. aeruginosa* 25 (26.3%) and *A. baumannii* 24 (25.3%) being the most common. This was followed by extended-spectrum beta-lactamase (*ESBL*) *E. coli* 22 (23.2%) and *K. pneumonia* 15 (15.8%). On the other hand, gram-positive bacteria were reported in 80 samples (84.5%), representing 18 different organisms, with *S. epidermidis* 17 (17.9%) and vancomycin-resistant *E. facium (VRE)* 17 (17.7%) contributing to the majority of infections, followed by *E. faecalis* in 7 (7.4%) patients. Furthermore, *C. albicans* 17 (17.9%) and *C. parapsillosis* 13 (13.7%) were the fungi that occurred most frequently in patients with nosocomial infections in the SICU. Table 3 represents the microbiological profile of nosocomial infections in the surgical ICU.

**Table 3:** Microbiological profile of nosocomial infections in the surgical intensive care unit

Organism	Total n (%) n = 157 (100%)*	With nosocomial Infection N = 95 (61%)*	Without nosocomial infection N = 62 (39%)
Gram-positive bacteria			
*S. epidermidis*	17 (10.8)	17 (17.9)	0 (0)
Vancomycin-resistant *E. facium*	17 (10.8)	17 (17.7)	0 (0)
*E. fecalis*	8 (5.1)	7 (7.4)	1(1.6)
*E. faecium*	7 (4.5)	7 (7.4)	0 (0)
*E. cloacae*	7 (4.4)	7 (7.4)	0 (0)
Gram-positive cocci, unspecified	6(3.8)	5(5.3)	1 (1.6)
Methicillin-sensitive *S. aureus*	3(1.9)	3(3.2)	0(0)
Methicillin-resistant *S. aureus*	3 (1.9)	3(3.2	0(0)
*Corynebacteria*	2 (1.3)	2(2.1)	0 (0)
*S. oralis*	3 (1.9)	3 (3.2)	0 (0)
*S. agalactiae*	2 (1.3)	2 (2.1)	0 (0)
*S. hemolyticus*	2 (1.3)	2(2.1)	0(0)
*Bacillus species*	2 (1.3)	2 (1.3)	0(0)
*Lactobacilli*	1 (0.6)	1(1.1)	0(0)
*S. capitis*	1 (0.6)	1(1.1)	0(0.0)
*S. hominis*	1 (0.6)	1(1.1)	0(0)
*S. pneumonia*	1 (0.6)	1(1.1)	0(0)
*Micrococcus luteus*	1 (0.6)	1(1.1)	0(0)
**Total**	**84 (53.3)**	**82 (85.8)**	**2 (3.2)**
**Gram-negative bacteria**			
*P. aeruginosa*	25 (15.9)	25(26.3)	0(0)
*A. baumannii*	25 (15.9)	24(25.3)	1(0.6)
*ESBL-E.coli*	23 (14.6)	22 (23.2)	1 (1.6)
*ESBL-K. pneumonia*	15 (9.6)	15(15.8)	0(0)
Gram-negative bacilli, unspecified	7 (4.5)	6(6.3)	1(1.6)
*CRE, unspecified*	6 (3.8)	6(6.3)	0 (0)
*E. coli*	4 (2.5)	4(4.2)	0 (0)
*Citrobacter*	3 (1.9)	3(3.2)	0 (0)
*M. morganii*	3 (1.9)	3(3.2)	0 (0)
*H. influenza*	2 (1.3)	2(2.1)	0 (0)
*Serratia marcescens*	1 (0.6)	1(1.1)	0 (0)
*P. mirabilis*	1 (0.6)	1(1.1)	0 (0)
*K. oxytoca*	1 (0.6)	1(1.1)	0 (0)
*Aeromonas*	1 (0.6)	1(1.1)	0 (0)
A. *Lwoffii*	1 (0.6)	1(1.1)	0 (0)
**Total**	**118 (74.9)**	**115 (121.4)**	**3 (4.8%)**
**Fungi**			
*Candida albicans*	17 (10.8)	17 (17.9)	0 (0)
*Candida parapsilosis*	13 (8.3)	13 (13.7)	0 (0)
*Candida glabrata*	7 (4.5)	7) 7.4)	0 (0)
*Candida tropicalis*	1 (0.6)	1 (1.1)	0 (0)
*Candida krusei*	1 (0.6)	1(1.1)	0 (0)
**Total**	**39 (24.8)**		
**No growth**	30 (19.1)	3(3.2)	27(43.5)

*total exceeds 100% as data are overlapping due to multiple microbiological profile of nosocomial infections

Regarding the utilization of antibiotics for these infections, 50 patients received vancomycin (52.6%), 26 patients received piperacillin/tazobactam (27.4%), and 35 patients received meropenem (36.8%). Table 4 shows the antimicrobials prescribed to patients with nosocomial infections.

**Table 4:** Antimicrobials prescribed for patients with nosocomial infections in the SICU

Antimicrobial	Total n (%) n = 157 (100%)*	With nosocomial Infection N = 95 (61%)*	Without nosocomial infection N = 62(39%)*
Vancomycin	73 (46.5)	50(52.6)	23(37.1)
Pipercillin/Tazobactam	38(24.2)	26(27.4)	12(19.4)
Meropenem	46 (29.3)	35(36.8)	11(17.7)
Fluconazole	23 (14.6)	14(14.7)	9 (14.5)
Colisitn	37 (23.6)	28(29.5)	9 (14.5)
Amikacin	35 (22.3)	29(30.5)	6(9.7)
Ceftazidime	25 (15.9)	21(22.1)	4 (6.5)
Metronidazole	24 (15.3)	14(17.4)	10 (16.1)
Levofloxacin	23 (15.3)	15(15.8)	9 (14.5)
Cefazolin	23 (14.6)	15(15.8)	8(12.9)
Ciprofloxacin	21 (13.4)	18 (18.9)	3 (4.8)
Cefuroxime	16 (8.9)	8 (8.4)	6 (9.7)
Gentamicin	14 (8.9)	12 (12.6)	2 (3.2)
Erythromycin	10 (6.4)	10(10.5)	0(0)
Clindamycin	11 (7)	9 (9.5)	2(3.2)
Cefotaxime	10 (6.4)	6 (6.3)	4(6.5)
Ceftriaxone	4 (2.5)	3 (3.2)	1(1.6)

*total exceeds 100% as data are overlapping due to multiple antimicrobials prescribed for patients

## Discussion

Nosocomial infections can spread in a variety of medical settings, including wards, surgical rooms, nursing homes, and others. There are numerous mechanisms by which infection occurs in the healthcare setting. In addition to contaminated tools and equipment, bedding, or aerosols, healthcare personnel can also spread illness [20]. The main objective of our study was to assess the incidence of nosocomial infections in SICU patients between 2020 and 2021.

The incidence of infections during stays in the ICU in Jenin, another West Bank district, in 2020 was 55% [21], while in Iran, it was 51.4% [22]. Both results were higher than the rate in our study, which included 352 patients, of whom 95 had either suspected or confirmed infections (26.9%) after staying in the ICU for more than 48 h. The incidence of nosocomial infections in our hospital was somewhat lower than the incidence found in India (33.3%) [8] and Boston City Hospital (31%) [8]. The overall mortality rate in our study was 34.4% in comparison with a study conducted in Libya in which the overall mortality rate was 29% [23] and a Chinese study in which the overall mortality rate was 23.6% [24]. The discrepancy between the values mentioned above is not inconceivable; many aspects must be considered, including patient demographics, ICU environment, admission diagnoses, type of surgery, and length of stay. Regarding suspected nosocomial infections, the uncertainty linked with early infection detection in critically ill individuals is well acknowledged because patients may display infection-related signs and symptoms due to noninfectious causes such as aspiration, venous thrombosis, and pancreatitis, for which even experienced intensivists struggle to appropriately identify infected patients who may benefit from early empiric therapy. Obviously, not all patients suspected of having infections are alike, and traditional objective measures of illness such as fever and leukocytosis cannot effectively distinguish between infected and uninfected patients. Therefore, improved diagnostic tools are necessary for rapid detection and differentiation of infectious from noninfectious causes [25]. Furthermore, in the intensive care unit, patients who are suspected of having an infection may not require antibiotics unless the infection is confirmed using a combination of laboratory, radiologic, and microbiological data, even if they are not in septic shock [25]. This approach can eliminate the reporting of nosocomial infections and the corresponding overuse of unnecessary antibiotics, as well as reduce collateral damage due to the emergence of multidrug-resistant organisms.

Pneumonia represented the highest percentage of all known sources of nosocomial infections in our study (36.8%), followed by skin and soft tissue infections (35.8%) and urinary tract infections (33.7%). However, the results of Baviskar et al. were not consistent with our study, as the most predominant cause of nosocomial infections in the study’s hospital ICU in India was skin and soft tissue infection (36.6%), followed by respiratory infections (24.4%) and genitourinary infections (23.4%) [8]. Pneumonia and UTIs were the most prevalent nosocomial infections in Gaza and Jenin, respectively [26]. Ventilator-associated pneumonia (VAP) and catheter-associated UTI were the predominant causes of infection in other countries [27].

The use of invasive medical devices is observed as a potential source of infection, especially in critically ill patients. By breaking down protective epithelial and mucosal barriers and favoring the growth and colonization of microorganisms in the different forms of foreign bodies introduced to the patient, the risk of device-associated infections is pertinent [28, 29]. The devices most frequently used in our SICU were endotracheal tubes (83.2%), urinary catheters (64.2%), and central lines (43.2%). A similar study of one-year duration in Libya showed comparable percentages of device-associated nosocomial infections, where endotracheal tubes (39.2%) and urinary catheters (19%) were considered the most common site of infection.

A great number of studies have reported the superiority of gram-negative organisms as a cause of NIs compared to gram-positive microorganisms [30]. In our study, 115 growths of the culture-confirmed infections were of gram-negative microorganisms, and 82 samples showed growths of gram-positive microorganisms. *P. aeruginosa* and *A. baumannii* were the microorganisms most commonly isolated in patients with nosocomial infections in the SICU, each comprising approximately 25 and 24 positive cultures, respectively, followed by *E. coli*. The most commonly remorted gram-positive organisms were *S. epidermidis* (17.9%) and *VRE* (17.7%). The results of our study were consistent with a 2-year prospective study carried out in the 15-bed ICU of Farawaniya Hospital in Kuwait, which showed that 68% of culture-confirmed pathogens were gram-negative species, 27% were gram-positive and 5% were fungi. The most prevalent organisms were *P. aeruginosa* (20, 17%), followed by *A. baumannii* (15, 13%), *Klebsiella* spp. (13, 11%) and *E. coli* (10, 8%) [31]. *A. baumannii* and *P. aeruginosa* are very often the cause of nosocomial infections in various hospital ICUs in different countries [32].

In our study, vancomycin (50, 52.6%), piperacillin/tazobactam (26, 27.4%), and meropenem (35, 36.8%) were the three drugs prescribed most frequently. In January 2005, a Turkish study showed that the most commonly used antibiotics were piperacillin/tazobactam, amikacin, and meropenem [33]. The prevalence of illness and death brought on by bacterial infections has significantly decreased because of the appropriate use of antibiotics. Nevertheless, the inappropriate utilization of these drugs has generated selective pressure and given rise to antibiotic resistance. Proper management of antibiotics in ICUs involves swift detection and effective treatment of bacterial infections in critically ill patients, as well as enhancing our capacity to prevent the administration of unnecessary broad-spectrum antibiotics, decreasing the length of their use, and limiting the number of patients who receive unnecessary antibiotic treatment [34, 35].

Fungi are not considered a familiar cause of nosocomial infections, but in our study, five strains of 39 fungi were isolated from patients in the SICU. The most frequently occurring Candida species was *C. albicans* (17), followed by *C. parapsilosis* (13), *C. glabrata* (7), *C. tropicalis* (1), and *C. krusei* (1). In January 2021, a study in China described eight species of Candida in 89 patients who acquired infections during their stay in a hospital ICU, of which six were attributed to *C. albicans* and two to *C. tropicalis* [24].

Healthcare-associated infections are known to prolong length of stay (LOS). Our study’s median duration of stay was 18.5 days. Meanwhile, in an Indian study, the average stay in the SICU was longer and equalled 14.4 days [24]. Extending the LOS by one day has been linked to the likelihood of raising the potential of acquiring an infection by 1.37%, while being infected also leads to an increase in LOS by 9.32 days. This leads to increased antibiotic use and promotes the development of antibiotic resistance, contributing to an increased financial burden on both the patient and the hospital [36].

In the ICU, patients are susceptible to hospital-acquired infections (HAIs), which can result in heightened morbidity and mortality. There is an increasing emphasis on the prevention of HAIs, and the implementation of infection control techniques is vital for addressing this concern. In recent times, various healthcare settings have witnessed progress in measures aimed at preventing infections. These measures encompass a focus on monitoring hand hygiene, revising isolation precautions, adopting novel approaches for environmental cleaning, implementing decontamination bathing, initiating antimicrobial stewardship programs, utilizing daily reassessment-intervention bundles, identifying and mitigating risk factors, as well as maintaining staff education initiatives and conducting active surveillance testing [37]. These efforts play a pivotal role in diminishing the occurrence of nosocomial infections [38, 39]. As demonstrated by several studies, strict adherence to meticulous infection control measures, particularly focusing on hand hygiene and robust implementation of evidence-based preventive techniques for ventilator-associated pneumonia and bloodstream infections, holds paramount importance in the reduction of NIs [40–45]. Our surgical ICU, situated within a bustling 6-bed unit at a tertiary care teaching hospital in the public sector, occupies a relatively compact space that lacks sufficient separation between the beds. Furthermore, our institution functions as an academic center with diverse medical and nursing specialties conducting clinical rotations throughout the year. These factors, indeed, have the potential to elevate the risk of NIs. Additionally, various investigations have unveiled that the utilization of invasive devices such as central venous or urinary catheters, intubation, tracheostomy, and mechanical ventilation, serves as a significant predisposing factor for infections [46, 47]. Therefore, the implementation of published and evidence-based infection control protocols is anticipated to substantially decrease the likelihood of pathogen transmission and the overall incidence of nosocomial infections.

### Strengths and limitations

Although this paper is one of the few studies conducted in Palestine that elucidate nosocomial infections in surgical ICUs, our research has several limitations. First, the data we collected were obtained from a single center and may not be generalizable to other centers. Second, our study was retrospective, and we were unable to identify the surveillance criteria necessary for identifying device-related infections, central line–associated bloodstream infections (CLABSIs), catheter-associated urinary tract infections (CAUTIs), ventilator-associated events (VAEs) and surgical site infections (SSIs), in addition to not representing the microbiological profile based on the isolation site.

## Conclusions

The incidence of suspected or confirmed nosocomial infections in all admitted patients to the SICU at An-Najah National University Hospital during the period 2020–2021 was 26.9%, and approximately 60.5% of the patients who received antibiotics during this period were confirmed or suspected to have nosocomial infections. Pneumonia, followed by skin and soft tissue infections and urinary tract infections, made up the great majority of infections. Gram-negative bacteria constituted the majority of reported cultures. Piperacillin/tazobactam and vancomycin were the most common antibiotics used to treat these nosocomial infections. We recommend that all healthcare workers in ICU departments strive for better strategies to minimize the incidence of nosocomial infections. This can be achieved by practicing hand hygiene, environmental hygiene, surveillance cultures, antibiotic stewardship programs, and following guidelines and patient safety cultures.

## Data Availability

Data and materials used in this work are available from the corresponding author upon request.

## List of abbreviations

ICU: intensive care unit
SICU: surgical intensive care unit
NNUH: An-Najah National University Hospital
NI: nosocomial infection
UTI: urinary tract infection
HAI: healthcare-associated infection
BSI: bloodstream infections
RSI: respiratory tract infection
SSI: surgical site infection
SSTI: skin and soft tissue infection
ESBL: extended-spectrum beta lactamase
VRE: vancomycin-resistant enterococci
NI: nosocomial infection
CDC: Centers for Disease Control and Prevention
VAP: ventilator-associated pneumonia
CAUTI: catheter-associated urinary tract infection
VAE: ventilator-associated event
LOS: length of stay
